# African Swine Fever Virus Interaction with Host Innate Immune Factors

**DOI:** 10.3390/v15061220

**Published:** 2023-05-23

**Authors:** Ayoola Ebenezer Afe, Zhao-Ji Shen, Xiaorong Guo, Rong Zhou, Kui Li

**Affiliations:** 1 Shenzhen Branch, Guangdong Laboratory of Lingnan Modern Agriculture, Key Laboratory of Livestock and Poultry Multi-Omics of MARA, Agricultural Genomics Institute at Shenzhen, Chinese Academy of Agricultural Sciences, Shenzhen 518000, China; afeayoola@gmail.com (A.E.A.);; 2 State Key Laboratory of Animal Biotech Breeding, Institute of Animal Science, Chinese Academy of Agricultural Sciences, Beijing 100193, China; 3 Guangdong Provincial Key Laboratory of Animal Molecular Design and Precise Breeding, Foshan University, Foshan 528231, China

**Keywords:** ASF, ASFV, virus virulence, pathogenesis in natural host, protective host immune response

## Abstract

African swine fever virus (ASFV) adversely affects pig farming owing to its 100% mortality rate. The condition is marked by elevated body temperature, bleeding, and ataxia in domestic pigs, whereas warthogs and ticks remain asymptomatic despite being natural reservoirs for the virus. Breeding ASFV-resistant pigs is a promising solution for eradicating this disease. ASFV employs several mechanisms to deplete the host antiviral response. This review explores the interaction of ASFV proteins with innate host immunity and the various types of machinery encompassed by viral proteins that inhibit and induce different signaling pathways, such as cGAS-STING, NF-κB, Tumor growth factor-beta (TGF-β), ubiquitination, viral inhibition of apoptosis, and resistance to ASFV infection. Prospects for developing a domestic pig that is resistant to ASFV are also discussed.

## 1. Introduction

Pork is an economically important protein source and a pivotal contributor to the livestock industry, generating significant revenue and employment opportunities. However, diseases in the pork industry have a substantial economic impact, causing low productivity, increased production costs, trade restrictions, and consumer concerns. The African swine fever virus (ASFV) has an increasing economic impact worldwide. Almost 300 million pigs have been lost to the disease or eliminated as a preventative measure since they were first detected in China in 2018. Consequently, businesses and economies have lost approximately USD 100 billion [1,2]. This disease is characterized by high fever, hemorrhage, ataxia, bleeding, and depression in swine. However, despite the virulence of the virus, African *Ornithodoros* soft ticks and other species, such as warthogs and bushpigs, are long-term reservoir hosts without significant clinical signs.

ASFV is categorized as an Asfivirus genus member of the Asfarviridae family [3]. It is a cytoplasmic replicated virus that belongs to the group of nucleocytoplasmic large DNA viruses (NCLDV) [4]. Depending on the viral strain, the genome of ASFV can range from 170 kb to 190 kb in size, coding for 150–200 viral proteins [5]. ASFV encodes more than 160 proteins; however, some are non-essential for ASFV replication but play essential roles in evading and modulating the host defense response [6]. These proteins perform various functions, such as manipulating host immunity, regulating and producing interferon I, inflammatory cytokines, protein buildup, apoptosis, and autophagy [7]. Many of these proteins also inhibit interferon and host antiviral responses. ASFV primarily targets mononuclear cells, such as macrophages and dendritic cells, which play a vital role in host innate immunity [8]. The mononuclear phagocyte system plays a critical role in the activation of innate and adaptive responses. Therefore, ASFV incapacitates these cells by profoundly manipulating their defense functions in response to infection [6]. Understanding the mechanisms by which ASFV successfully modulates host defense responses to infection will provide insights into the strategies and knowledge required for the development of ASFV-resistant pigs via selective breeding by manipulating the interaction sites of the virus. The cyclic GMP-AMP synthase (cGAS) pathway, a crucial component of the innate immune response, is responsible for identifying viral infections and triggering antiviral reactions by activating the cGAS-STING pathway. An essential feature of the host defense, operating immediately upon infection with these viruses, is the capacity of the cell to induce intrinsic and innate immune responses against DNA viruses. These host defenses function to prevent viral infection, support adaptive immune responses within the host organism, and promote a global antiviral environment. These immune processes are essential for the survival of the host and for preserving a balanced system. Unfortunately, there is still a lack of knowledge regarding the essential cellular processes involved in ASFV infections. In addition, owing to the lack of a vaccine, the development of genetically engineered pigs to mitigate ASFV is conceivable using gene-editing techniques.

This review explores the mechanisms and proteins employed by ASFV to override its host innate defense systems, such as signaling pathways in the innate immune system that inhibit interferons, NF-κB, ubiquitination, and apoptosis. We also provide possible mechanisms for the development of ASFV-resistant pigs by manipulating the viral interaction sites. Exploring the critical signaling pathway in the host can be a path to successfully creating a pig resistant to the raving virus.

## 2. ASFV Proteins and Innate Immunity

The primary function of the immune system is to identify and destroy pathogens. It is important to note that type I interferons and effector cells, such as macrophages and dendritic cells, are crucial for initiating innate immune responses. The detection of pathogen invasion and danger signals through pattern recognition receptors (PRRs) is primarily influenced by macrophages and dendritic cells, which play a significant role in innate immunity. Pathogen-associated molecular patterns (PAMPs) are specific to microbes and are recognized by innate pathogen sensors called pattern recognition receptors (PRR). There are several classes of PRRs, such as C-type lectin-like receptors (CLR), RIG-like receptors (RLR), Toll-like receptors (TLRs), nucleotide oligomerization domain (NOD)-like receptors (NLRs), and cyclic GMP-AMP synthase (cGAS). All these receptors are essential for immunity because they recognize viruses and other pathogens and their associated molecular patterns [9,10,11]. One of the vital PAMP in viral particles is the nucleic acid that stimulates antigen-presenting cells (APC), leading to the activation of PRRs [9].

To circumvent the defense mechanisms of the host, such as the interferon system, apoptosis, and inflammation, ASFV has developed different evasion tactics to target critical signaling pathways such as cGAS-STING, NF-κB, tumor growth factor-beta (TGF-β), and ubiquitination [12], (Table 1). The activation of several secretory signals, including cytokines and chemokines, as well as the initiation of phagocytosis and inflammation, are examples of intercellular signals [9]. ASFV is extremely adept at evading the host immune system and establishing full infection. This makes ASFV a dangerous virus and a significant concern for the swine industry. However, regulation of the host’s innate immune system is essential for protection against ASFV infection. However, developments in this field have been hindered by inadequate knowledge of the roles of these proteins and the processes involved.

## 3. Cyclic GMP-AMP Synthase Signaling Pathway

Monomeric cGAS is located in the cytoplasm of healthy cells and lacks the ability to bind DNA or function as an enzyme [38]. DNA binding by cGAS initiates the synthesis of cyclic GMP-AMP (cGAMP). To continue the chain reaction, cGAMP functions as a second messenger by binding to and activating the STING adaptor, which recruits TANK-binding kinase 1 (TBK1) and interferon regulatory factor 3 (IRF3) [39,40]. Upon stimulation with cGAS-STING, the pathway causes a downstream signal transduction cascade involving TANK-binding kinase 1 (TBK1) and interferon regulatory factor 3 (IRF3) and its translocation to the nucleus. The cGAS/STING signaling pathway has gained prominence as an essential immune regulator in response to pathogens [41]. The role of cGAS in detecting cytosolic or viral DNA has been the subject of extensive research [42]. This pathway induces the production of type I interferons and other pro-inflammatory cytokines, ultimately leading to an effective antiviral immune response [15,18,40].

**Figure 1:**
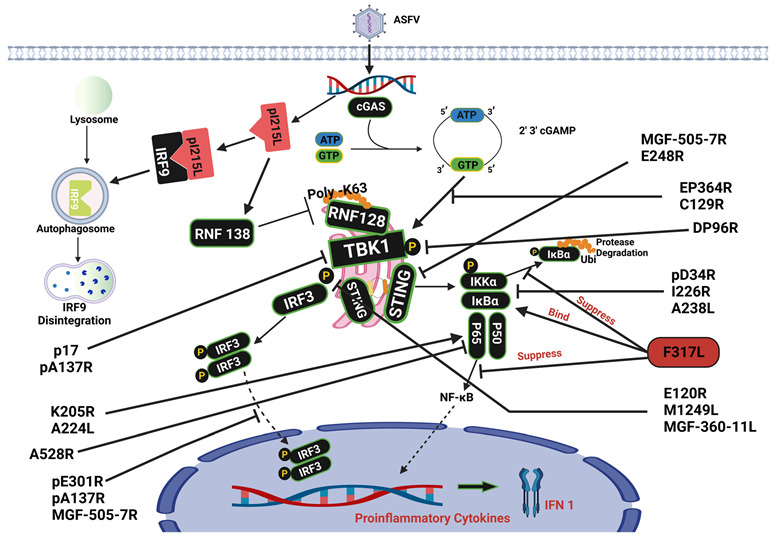
ASFV proteins modulating the cGAS/STING and NF-κB signaling pathway (Created with BioRender.com).

Stimulation and manipulation of the production of immunomodulatory molecules by ASFV have been reported in many cases (Figure 1). Several ASFV proteins modulate the cGAS-STING pathway and aid infection. Several studies have reported that ASFV inhibits the cGAS-STING signaling pathway [7,24]. ASFV proteins C129R and EP364R interact with cGAS and cleave 2′,3′-cGAMP, thus disrupting cGAS-STING signaling and inhibiting IFN responses [13]. pE301 negatively regulates the cGAS-STING pathway by preventing nuclear import of IRF3, which results in the inhibition of IFN-I synthesis and undermines the host’s innate immune system against the virus [7]. The protein acts as an antagonist with multiple inhibitory roles, as reported by Liu et al. (2022); pe301r inhibits the activities of NF-κB and interferon-beta (IFN-β), which were enhanced by cGAS-STING. The repressive actions of pE301R have also been shown to occur downstream of interferon regulatory factor 3 phosphorylation. By interacting with IRF3, pE301R prevents IRF3 nuclear translocation [7]. This is also achieved by ASFV virulence factor pA137R, which promotes TBK1 destruction, thus decreasing IRF3 translocation. In both cases, the inability of IRF3 to access the nucleus inhibits the activation of IFN transcription [14]. It has also been observed that ASFV DNA substantially inhibits the cGAS-STING signaling pathway by utilizing structural proteins such as M1249L. The ASFV proteins M1249L and DP96R decrease IRF3 levels and inhibit TBK1 phosphorylation [43], inhibiting type I interferon synthesis [15]. M1249L, a viral protein found in the capsid, decreased TBK1 phosphorylation and significantly suppressed the action of the IFN-β promoter enhanced by cGAS-STING. A subsequent study revealed that M1249L colocalizes and induces IRF3 breakdown via the lysosomal channel [15]. Another ASFV structural protein, p17, prevents the cGAS-STING cascade by binding to STING to prevent the induction of TBK1 and inhibit the IKK complex [16]. The highly pathogenic strain Armenia/07 interferes with the cGAS-STING signaling pathway, which impairs the activation of STING [44] and prevents the generation of IFN-β [44,45].

ASFV’s virulence depends on its capacity to inhibit IFN synthesis [24]; another inhibitor of IFNβ production is E248R, an ASFV protein that interacts with and inhibits STING. Overall, the E248R protein suppresses the innate immune response of the host by preventing STING expression [36]. ASFV proteins manipulate the synthesis of host immunomodulatory molecules. The structural ASFV protein E120R interacts with IRF3 and interferes with the activation of IRF3 by TANK-binding kinase 1 (TBK1), which in turn suppresses IRF3 phosphorylation, decreasing interferon production. Deletion of two positions in E120R results in disruption of E120R-IRF3 interaction, and a virus harboring the deletions induces more robust IFN responses in porcine alveolar macrophages (PAM) than the wild-type virus [18]. In swine alveolar macrophages derived from specific pathogen-free Landrace pigs, ASFV HLJ/18 infection results in reduced type I IFN production and suppressed type I IFN production triggered by cGMP-AMP [24].

Multiple members of the multigene families, MGF360 and MGF505, play a role in controlling interferon activation. The data reported by Yang et al. (2022) showed that ASFV MGF360-11L significantly inhibited IL-1β, IL-6, and IFN-β secretion in PAM cells upon ASFV infection by downregulating cGAS-STING. MGF360-11L achieved this by blocking the phosphorylation of TBK1 and IRF3 induced by cGAS-STING [17]. Interactions of MGF505-7R with IRF3 prevented the translocation of IRF3 to the nucleus. When this occurs, proinflammatory cytokine synthesis and IFN-β output decrease [11]. MGF505-7R reduces STING expression by increasing the production of ULK1, a protein involved in autophagy [11]. MGF binds to STING and promotes its degradation through lysosomal and autophagic mechanisms [19]. ASFV MGF family members indirectly control IFN-β synthesis, which negatively regulates the innate immune response. Interferon therapy can significantly reduce ASFV multiplication, both in vivo and in vitro [40], highlighting the central role of interferon in the pathophysiology of ASFV.

It is widely acknowledged that cGAS-STING-mediates antiviral responses [22]; however, ASFV evades the host’s innate immunity mechanism. Nevertheless, the various molecular pathways by which ASFV proteins adversely regulate the cGAS-STING signaling cascade, such as type I IFN (IFN-I) synthesis, remain poorly understood [7]. ASFV modulates the cGAS/STING pathway with a major focus on inhibiting TBK1 phosphorylation; these affect the downstream genes, such as the inhibition of IRF3 and interferon synthesis.

## 4. NF-κB Signaling Pathway

The nuclear factor kappa B (NF-κB) group is a highly conserved group of transcription features associated with regulating proinflammatory cytokines and antiviral genes, which are crucial for both innate and adaptive immune responses to viral infections [46]. This group consists of the following related associates: p50 (NF-κB1), p52 (NF-κB2), p65 (RelA), RelB, and c-Rel, which bind to the κB promoter, a particular DNA region, and regulate target gene transcription [46,47].

When the pattern recognition receptor (PRR) is activated by a viral particle, signal transduction activates IKK combinations (made up of IKK-α, -β, and-γ), which phosphorylate IκB molecules, resulting in their disintegration, as well as the liberation of unbound NF-κB subunits, which then activates nuclear translocation. In the nucleus, NF-κB initiates the production of pro-inflammatory cytokines with adverse feedback cycles to terminate the inflammatory reaction [48]. There have been discoveries regarding the molecular processes that link the NF-κB signaling pathway with ASFV infection. ASFV proteins regulate NF-κB, as reported in several studies. ASFV infection severely compromises the immune system; however, the understanding of the underlying systems is limited. It has been established that ASFV can modify NF-κB signaling to decrease the production of cytokines that promote inflammation and enhance the proliferation of the virus. The pD345L protein suppresses NF-κB signaling by inhibiting the activity of IKK kinase [20]. During viral infection, ASFV I226R suppresses the stimulation of NF-κB and IRF3, thereby hampering antiviral responses [21]. This protein is believed to use several mechanisms to counteract innate immune responses, such as the disintegration of NF-κB essential modulator (NEMO) [21], ultimately impeding the host’s natural defense mechanisms against the virus.

ASF viral protein F317L suppresses NF-κB stimulation to prevent host immune reactions. F317L binds to IκB kinase β (IKKβ) and hinders its ability to undergo phosphorylation, which in turn decreases IκBα ubiquitination and phosphorylation and increases IκBα stability [22]. Blocking NF-κB initiation, thereby preventing nuclear translation, leads to the buildup of IκBα and reduces the production of several cytokines that promote inflammation. F317L is a crucial ASFV protein that disrupts the NF-κB activity. Examination of the terminated mutations showed that amino acids 109–208 in F317L are essential for NF-κB suppression [22]. Exploring the interaction domains or locations of IKkappa utilized by F317L is necessary to understand how it regulates its targets. ASFV pS273R, a small ubiquitin-like modifier-1 (SUMO)-specific protease, has been reported to manipulate and reduce cGAS-STING signaling. pS273R interferes with the interaction between IKKε and STING [37] and also obstructs pyroptosis [49].

ASFV A528R has been shown to decrease IFN-β induction by targeting NF-κB [23]. According to previous studies, A528R can interrupt the TLR8-NF-κB pathway by inhibiting downstream promoters, phosphorylation of NF-κB p65, nuclear translocation, and antiviral activity. ASFV utilizes MGF proteins to evade NF-κB. pMGF505-7R suppressed NF-κB activity by binding to IKKα, thereby preventing its translocation to the nucleus [11]. The inhibitor of NF-κB prevents the expression of the phosphorylated forms of NF-κB p65 and p-IκB proteins [50], thereby preventing IL-1β production. UBCv1 has also been identified as a new inhibitor of NF-κB activation, which hinders the translocation of p65 into the nucleus [51]. Silk et al. (2007) revealed that ASFV A238L blocked the NF-κB pathway. The protein was discovered in both the nucleus and cytoplasm of cells that had been infected with ASFV [34]; however, the deletion of A238L did not eliminate ASFV infection [52]. In addition, triggering the NF-κB promoter by cGAS/STING, TBK1, and IKKβ was explicitly inhibited by DP96R [43].

Not every ASFV protein encodes NF-κB inhibition; a protein similar to IAP obstructs caspase-3 activation and improves cell viability encoded by ASFV. ASFV IAP A224L stimulates NF-κB transcriptional activity [27,53]. K205R stress stimulation in the endoplasmic reticulum (ER) chamber leads to P65 being propelled into the nucleus, activating NF-κB [26]. The hallmark of the proliferation of ASFV progeny by evading the host’s immune responses has been its ability to interact and manipulate the IKK complex, which is responsible for phosphorylation, degradation of IκBα, and translocation of NF-κB to the nucleus to activate gene expression.

## 5. Transforming Growth Factor-β Signaling Pathway

All porcine cells generate transforming growth factor-β (TGF-β), which plays a crucial role in mammalian homeostasis and growth. TGF-β occurs in three forms (TGF-β 1, 2, and 3), all of which operate as receptor ligands and play critical roles in controlling cell proliferation and apoptosis [54]. TGF-β induces TGFbRII activation, which, in turn, phosphorylates TGFbRI and initiates signal transduction. This signal causes receptor-regulated SMADs to become phosphorylated and bind to Co-SMADs, activating the SMADs. It then moves into the nucleus, where it regulates the transcription of specific genes [55]. TGF-β plays a pivotal role in the peripheral T-cell balance [56]. It has the same effect on macrophage polarization in mammals, shifting them to an anti-inflammatory phenotype [57]. Although the fundamental molecular mechanisms of TGF-β have been defined in great detail over the past several years, the elements that affect TGF-β response remain unclear [58,59]. Many viruses play important roles as stimulators of the transforming growth factor-beta pathway [55]. Anti-inflammatory mechanisms include TGF-β stimulation [60]. According to Kowalczyk et al. (2015), viral products induce TGF-β mRNA expression by binding to certain receptors. The presence of a highly pathogenic strain of ASFV leads to the secretion of this cytokine. O’Donnell et al. (2017) [61] examined TGF-β levels on days 0, 7, and 14 after immunization of pigs with ASFV-G-9GL/UK, an attenuated mutant. A few of the studied animals exhibited elevated TGF-β levels after immunization. Therefore, ASFV induces TGF-β synthesis in infected macrophages and inhibits the synthesis of IL-8, IFN-α, and TNF-α [62]. The clinical results, as well as hematological parameters of swine at 12 and 18 weeks old infected with the Netherland’86, an isolate with a mildly pathogenic strain, and their reactions, were compared. However, during the investigation period, TGF-β concentrations in the serum were constant between 0 and 27 days of Pi treatment [63]. The continuous upregulation of almost all critical proinflammatory cytokines implicated in viral infection was elicited by virulent ASFV SY18, as IL-10 and TGF-β cytokines with anti-inflammatory properties were not [64]. TGF-β and IL-10 enhance anti-inflammatory mechanisms in macrophages. Franzoni et al. (2022) studied how immune-suppressing cytokines affect macrophage reactions and their susceptibility to infection using two strains of ASFV. The effects of TGF-β and IL-10 on the infectivity of swine monocyte-derived macrophages (moMΦ) were investigated. This study demonstrated that neither IL-10 nor TGF-β pretreatment influenced cytokine responses to NH/P68 and 26544/OG10 ASFV strains [5,65]. There is insufficient research examining the impact of ASFV proteins on this versatile cytokine; further investigation of the role of TGF-β in modulating immune responses and protecting against ASFV infection is required.

## 6. Ubiquitination

Ubiquitination is a post-translational alteration that causes the attachment of ubiquitin to its substrates [66,67]. Its function in any of the following formats may move proteins to different subcellular locations, change their activities, facilitate or block their reaction with other proteins, and mark them for disintegration via the proteasome [68]. Viruses have evolved methods for regulating translation in host cells to facilitate viral protein production. ASFV proteins have been shown to modulate ubiquitination, increase viral replication, and inhibit the host immune response. This action is significant to the virus because ubiquitination regulates several important cellular functions, such as protein degradation, signaling, and DNA repair.

One of the most potent mechanisms suppressing type I IFN production is the viral E2 ubiquitin-conjugating enzyme ASFV [24,69]. ASFV has developed a variety of strategies to resist type I interferon (IFN) and other immunomodulatory proteins, which are a decisive part of the innate antiviral response and enhancement of infection [24,69]. The I215L gene of ASFV encodes a ubiquitin-conjugating enzyme, making it the first virus of its kind [70]. ASFV pI215L stimulates the engagement of RNF138 and RNF128, leading to the elevation of RNF138 to disintegrate RNF128 and prevent K63-linked ubiquitination of tank-binding kinase 1 (TBK1) [24,71]. Polyubiquitination of pI215L was observed rather than multiple mono-ubiquitination [69]. The innovative purpose of pI215L is to act as an inhibitor of type I interferon signaling [70]. The clever immune evasion tactic developed by ASFV also showed that pI215L destroys host interferon regulatory factor 9 (IRF9) through the autophagic route, preventing type I IFN initiation and thwarting innate immune reactions in the host. Due to the disintegration of interferon regulatory factor 9 (IRF9), viral pI215L suppresses the activity of the promoter of IFN-stimulated response elements (ISREs) and the transcription of IFN-stimulated genes (ISGs). Mutated dormant pI215L maintains the capacity to suppress type I IFN signaling, suggesting that the breakdown of IFR9 is caused by ASFV E2 ubiquitin through a process that does not rely on ubiquitin-conjugating activity [70]. Silencing pI215L activity stifled the spread of ASFV but also stimulated the synthesis of interferon beta (IFN-β) [24]. Further studies on pI215L and its functions, such as its interaction with RNF138 and its capacity to degrade other molecules, such as RNF128, via K48-linked polyubiquitination, are needed.

ASFV utilizes numerous proteins to trigger autophagy disintegration; pA137R binds to TBK1 and causes its degradation through lysosome-mediated autophagy [14]. Other ASFV proteins, such as MGF-505-7R [72], E199L [25], and K205R, also initiate autophagy [26]. However, this might reflect a method shared by several virulence genes to control the host’s innate immunity triggered by ASFV.

## 7. ASFV Modulating Apoptosis Protein

**Figure 2:**
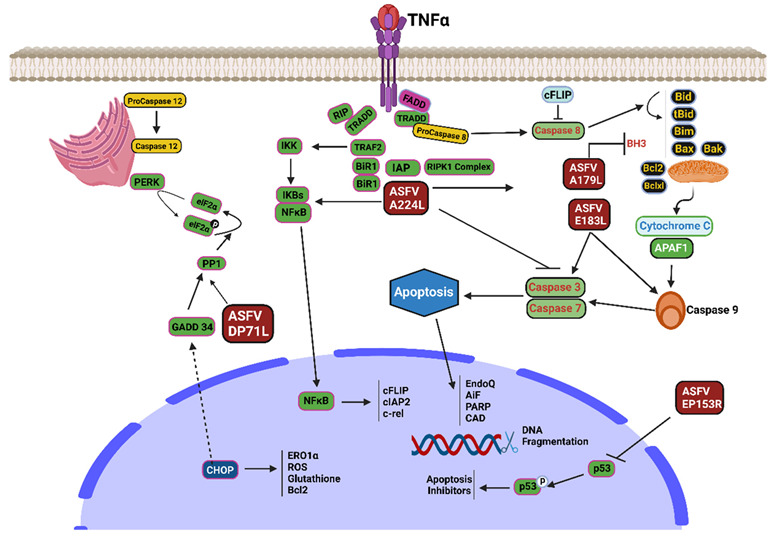
ASFV proteins mechanisms for modulating apoptosis (Created with BioRender.com).

Viruses can adapt to the host species because they are molecular deceivers. The ability to bypass host defenses is crucial for viral persistence, reproduction, and spread [73]. Many mammalian host defense mechanisms involve apoptosis of the infected host cells. Preventing the virus from finishing its viral replication decreases the quantity of harmful progeny produced. Since apoptosis of infected cells effectively prevents viral proliferation [73], ASFV has developed complex molecular mechanisms to overcome host defenses. ASFV has evolved pathways in infected cells to prevent apoptosis (Figure 2). As a cause of infection, ASFV hijacks numerous host defense mechanisms, leading to instantaneous cell death via regulatory mechanisms such as necrosis, pyroptosis, and apoptosis [53].

## 8. Inhibition of Apoptosis

### 8.1. A179L Protein

The B-cell lymphoma-2 (Bcl-2) family governs apoptosis triggered within the cells or mitochondria. Proteins associated with Bcl-2 are critical regulators of the discharge of apoptotic regulators from the mitochondria, as they are the primary deciders of mitochondrial viability [28]. Bcl-2 family members that prevent cell death (anti-apoptotic) possess several BH (Bcl-2 homology) domains that range from BH1 to BH4, whereas Bcl-2 relatives that promote cell death (proapoptotic) have numerous BH domains, which also lie within BH1 to BH3 or just the BH3 domain [74]. Apoptosis is initiated in response to cellular injury and infection. BH3-only proteins signal the execution of the programmed cell death pathway, at which point the proapoptotic proteins Bax and Bak permeate the outer mitochondrial membrane [53,75]. Viruses have adapted methods to suppress apoptosis during the earliest phases of infection, thereby protecting themselves from host cell mortality as they begin to proliferate. Viral proteins that act similarly to the mammalian anti-apoptotic Bcl-2 proteins are responsible for this phenomenon. Numerous vBcl-2 homologs share structural similarities with their mammalian Bcl-2 orthologs, including the characteristic Bcl-2 α-helical fold [74]. The A179L protein is an effective anti-apoptotic agent [27], similar to Bcl-2, and is an immunoregulatory and pathogenic ASFV-encoded protein.

Evidence suggests that A179L binds multiple apoptosis-inducing Bcl-2 proteins. Nevertheless, the interaction hierarchy and structural foundation underlying the prevention of apoptosis remain unclear. A systemic foundation for the hindrance of apoptosis was obtained by determining the crystalline assembly of the A179L protein in a multifaceted manner with the BH3 domains of Bid and Bax [28]. ASFV A179L inhibits apoptosis caused by viral infection while promoting necroptosis by engaging the BH3 motif of the proapoptotic Bcl-2 gene family through a unique binding affinity cleft.

Necroptosis and apoptosis are more likely to occur because of the ASFV virus. Hernaez et al. (2004) reported that the activation of caspases 9 and 3 by the ASFV protein E183L or p54 is responsible for initiating necroptosis [76]. The activation of apoptosis is initiated by this protein, which was the first identified component of ASFV to do so. Shi et al. (2021) showed that A179L boosts necroptotic cell counts in TSZ-treated L929 cultures and facilitates TNF-α phosphorylation. In a pig intestinal epithelial cell line infection model, A179L inhibited apoptosis by inhibiting the cleavage of PARP, caspase 8, and caspase 3 [29].

### 8.2. A224L Protein

IAPs are a group of proteins that prevent programmed cell death by binding to and deactivating caspases 3, 7, or 9 [77]. ASFV encodes a protein similar to IAP that blocks the activation of caspase-3 and enhances the ability of cells to survive [53]. When cells expressing A224L are stimulated by phorbol myristate acetate (PMA) [53], there is an increase in NF-κB activity [50], thereby leading to an increase in the expression of anti-apoptotic genes.

### 8.3. EP153R

EP153R, a non-essential protein with a C-type lectin domain encoded by the ASFV ORF, has been found to prevent apoptosis by activating the p53 and caspase 3 pathways. It is expressed during the initial and final phases of the viral lifecycle. EP153R decreases the transactivating ability of p53 in Vero cells due to virus-induced cell death. It is possible to understand the process of EP153R stimulation by noting that p53 induces transcription of different apoptosis antagonists [31]. Ca^2+^ glycan proteins, which are crucial for cell-to-cell adhesion and are thus required for C-type lectin function, are essential for adaptive and innate immunological responses [32]. EP153R inhibits MHC-I exocytosis and decreases p53 activity. It also engages with MHC-I through its lectin region. Petrovan et al. (2022) investigated the role of EP153R in lowering ASFV perseverance and virulence in pigs infected with BeninΔDP148R.

### 8.4. DP71L

ASFV DP71L inhibits the synthesis of proteins that promote stress-induced apoptosis. Viral infections cause cellular stress, such as endoplasmic reticulum (ER) stress, which stimulates the unfolded protein response (UPR). DP71L utilizes protein phosphatase 1 to dephosphorylate translation initiation factor 2α (eIF2α) and prevents overall protein expression and deactivation of the pro-apoptotic CHOP factor [33]. However, some viral proteins might have complementary activities to carry out a specific function, as Zhang et al. (2010) [78] reported that the knockout of the DP71L gene does not cause a boost in eIF-2α phosphorylation or CHOP initiation. ASFV DP71L was also reported to prevent the expression of UPR-associated molecules such as CHOP [79].

## 9. *Ornithodoros* Tick Antiviral Response to ASFV

The sylvatic cycle between warthogs and soft ticks infesting their burrows leads to the persistence of ASFV in sub-Saharan Africa [80], which renders ASFV a solitary arthropod-borne virus with DNA [81]. Ticks of the *Ornithodoros* genus, particularly those belonging to the species *Ornithodoros moubata*, have a crucial impact on the transmission of ASFV, among the numerous factors that affect its dissemination. [82]. Certain species of *Ornithodoros* soft ticks can transmit ASFV [83], but *O. erraticus* and *O. verrucosus* were unsuccessful in transmitting Eurasian strains of the virus [84]. Soft ticks infected with ASFV can preserve the virus for extended periods [85]. However, relative to the widespread transmission of the virus globally, it is evident that soft ticks do not significantly contribute to the current epidemic because they are not actively involved in disseminating the virus over long distances. However, this has not been the subject of much debate. Tick tissues provide an environment allowing the virus to replicate [85], and laboratory testing results suggest that ASFV can survive within *O. moubata* for a maximum of three years, utilizing multiple modes of transmission, including transstadial, transovarial, sexual, and direct-to-animal transfer [82].

Despite the virulence of ASFV, warthogs and ticks are natural reservoirs that remain resistant and show no symptoms of infection. There are several plausible explanations, but the reasons for their tolerance to ASFV remain unknown. Nevertheless, innate immune signaling pathways prompt antiviral responses in ticks. Arthropods, including ticks, primarily use RNA interference (RNAi) to fight viral infection [86]. Although RNAi is a vital aspect of antiviral response, research has indicated that other innate immune pathways also play a role in managing infections in arthropod vectors. Specifically, the JAK/STAT, Toll, and Imd pathways are associated with antiviral defense in insects [87]. In addition, the tick immune system may be more effective in recognizing and neutralizing viruses than the pig immune system. Ticks have evolved to feed on the blood of many hosts, including birds, reptiles, and mammals, and their immune systems have adapted to deal with a wide range of pathogens. This may provide them with an advantage in dealing with ASFV infection. Additionally, through saliva, ticks can suppress the host’s innate immune response, complement system, and adaptive immunity [88]. Moreover, some tick species produce saliva that contains macrophage migration inhibitory factors (MIF), which can hinder the migration of macrophages [89]. Tick saliva can decrease or increase the production of pro-inflammatory and anti-inflammatory cytokines, thereby inhibiting inflammation. Hyalomin-A and hyalomin-B are two proteins found in tick saliva that have significant anti-inflammatory effects, reducing the secretion of specific cytokines, such as C–C motif chemokine ligand 2 (CCL2), tumor necrosis factor-alpha (TNF-α), and interferon-gamma (IFN-γ), and increasing the production of interleukin -10 [89].

Although the exact mechanisms of innate immunity in *Ornithodoros* ticks against ASFV are not yet fully understood, ticks have evolved several defense mechanisms to limit viral replication and transmission.

## 10. Warthog Resistance to ASFV

ASFV affects domestic pigs; however, warthogs are a natural reservoir of the virus, yet they are naturally immune to it and do not show any symptoms of the disease [90]. Recently, researchers have focused on the mechanisms that contribute to ASFV resistance in warthog and other species. The host and the virus factor out of several issues become essential. The types of hosts and their innate immune systems determine the etiology of ASFV [80].

The genetic makeup between a warthog and a domestic pig determines the differences in each host’s reaction to ASFV infection. Several mutations have likely shaped the genomes of warthog in response to natural selection pressures, including the loss of genes and the reduction or growth of particular gene families. Comparing the warthog and pig genomes, Feng et al. (2021) reported that the lactate dehydrogenase B (LDHB) gene on pig chromosome 2 was lacking in the genome of warthogs.

The role of NF-κB in immunomodulatory activity during viral infection is vital to the innate immune response. Stimulating NF-κB can enhance the production of antiviral cytokines and other immune effector molecules, which can help restrict viral proliferation. RELA is associated with ASFV resistance. Inherent heterogeneity in RELA, the variability of activity of NF-κB, the RELA gene that codes for vital elements in the NF-κB signaling pathway, contains 15 nucleotide variations between domestic pigs and warthogs, leading to 3 amino acid variations [91]. Evidence from investigations of mutations suggests that the S531P site accounts for the vast bulk of the difference in values between warthogs and domestic pig RELA. The differences in RELA between warthogs and pigs may explain the different tolerance levels of these two species [91]. Resistance to ASFV may be due to variances in the induction of the nuclear factor–kappa B pathway; this should be investigated in future studies. NF-κB is a critical player in the innate immune response and is composed of transcription elements that regulate the production of many cytokines involved in inflammation and proteins that resist cell death [46]. The ReLA in domestic pigs was modified by inserting NF-κB motifs from warthogs. Even though some animals showed delayed onset of clinical symptoms and lower viral load of ASFV DNA in blood tests and nasal secretions, this information is insufficient to confer resistance to infection from ASFV [92]. Therefore, warthogs may be less susceptible to ASFV NF-κB inhibitors. Based on results from reporter studies performed on mouse embryonic fibroblasts, it was observed that the domestic pig RELA subunit (NF-κB) is more basally active but less responsive to stimulation than its warthog counterpart [91], and the S531P mutation in warthog may be responsible for the observed difference. Although genetic resistance to ASF viral infections has been demonstrated, phenotypic evidence supporting this finding requires further investigation.

Host resistance may be due to the possibility that the innate immune response is better able to prevent pathogenic responses and limit viral replication in hosts that do not become ill. Lessening of clinical symptoms may also be attributable to host endurance and genetic variables that inhibit the over-activation of virulence reactions [91,92].

Furthermore, because the degree of viral virulence varies widely across warthogs and domestic pigs, it is likely that the innate immune response plays a crucial role in these processes. Therefore, the innate immune system may prevent immune response and efficiently regulate virulence in asymptomatic hosts. The possible host and viral variables were excluded. For instance, ASFV genetic variables may be less efficient in regulating innate reactions in warthogs than in domestic pigs. Host factor variables might even mitigate detrimental reflex over-activation, making host tolerance a possible explanation for attenuated clinical manifestations.

Other factors, such as the severity of the viral variant and other pre-existing underlying diseases, may also contribute to the susceptibility of animals to ASFV. However, warthogs may also have a higher degree of resistance to the virus because of their robust immune systems and exposure to various pathogens in their natural habitats. However, it is challenging to attribute this to any factor because of the absence of accessible experimental and genetic evidence.

## 11. Discussion and Future Perspective

ASFV interferes with the host’s innate immunity by manipulating the host cytokine levels through the pathways mentioned above. Viral proteins released during ASFV infection form a network interactome, potentially stimulating or suppressing the cGAS-STING signaling pathway. While most studies have focused on the cGAS/STING pathway, some DNA sensors, such as TLR9, may be feasible for detecting ASFV. However, RNA sensors may also detect ASFV. Further research is encouraged on the different proteins in ASFV that modulate host immune factors, such as interferon regulatory factors 3 and 9, TGF-β, and NF-κB.

Disease-resistant animals have been the focus of selective breeding programs. To develop ASFV-resistant pigs, genetic markers can be used to distinguish between pigs that are vulnerable and resistant to ASFV. Breeding of ASFV-resistant pigs may be achieved by genetic engineering, a technique already utilized to create virus-resistant pigs against diseases such as transmissible gastroenteritis virus (TGEV), classical swine fever virus (CSFV), and porcine respiratory and reproductive syndrome virus (PRRSV). Xu et al. (2020) [93] modified the alleles of knockout genes for the receptor proteins CD163 and pAPN to produce pigs with double-gene knockout (DKO). These DKO pigs were shown to have complete protection against PRRSV and TGEV even when exposed to the virus. Somatic nuclear transfer presents an opportunity to create transgenic (TG) pigs that are resilient to CSFV using an RNA interference technique coupled with CRISPR/Cas9 [94]. CRISPR-Cas9 technology has also been tested to genetically engineer CD163 to produce pigs that are immune to PRRSV [95,96]. It was hope coming through when Sánchez-Torres et al. (2003) [97] reported that the receptor for ASFV might be CD163. Georgia 2007/1 was used as the inoculum in a study on macrophages that had CD163 completely knocked out. The wild-type and knockout caught the infection. According to these data, Georgia 2007/1 strain infection of ASFV does not depend on CD163, and the data also suggest that CD163 does not play a major role in ASFV infection [98]. Research has shown that animals may simultaneously resist two important viruses through multiple gene knockouts, as Xu et al. (2020) [93] reported. Nevertheless, further studies are needed to determine whether the simultaneous silencing of numerous potential ASFV receptor genes may achieve ASFV resistance, although the specific receptors(s) and attachment factors utilized by ASFV remain unclear. Furthermore, a new direction can be proposed for genetically manipulating the viral interaction sites. Genetic manipulation of viral interaction sites with the host’s innate immunity could be a future solution for achieving ASFV-resistant pigs. One of the first steps in mounting an antiviral defense system is the activation of cGAS, a crucial cytosolic DNA sensor. An improved host immune response to ASFV infection can also be achieved by genetically increasing cGAS-STING activity in pigs. Therefore, the host immune reaction to ASFV infection can be improved by increasing the activity of its favorable regulators, decreasing the activity of negative regulators, or adjusting the activity of the downstream effectors in the cGAS-STING cascade. An increase in downstream effectors creates a robust antiviral status that can lead to increased host survival and decreased viral transmission. The genetic manipulation of warthog DNA in the domestic pig genome should be examined again. Even though the modification of the domestic pig RELA with warthog failed, a change in dimension to interaction sites can be experimented with.

Conclusively, various ASFV proteins affect different stages of the cGAS-STING pathway, inhibiting the production of IFN. Therefore, developing drugs targeting multiple viral proteins and applying them in animal husbandry seems impractical. Studies have shown that interrupting the transport of STING from the Golgi to the lysosome and prolonging STING’s residence time in the Golgi can recruit more TBK1 and IRF3 and phosphorylate them, resulting in higher levels of IFNβ production in mouse embryonic fibroblasts with the knockout of the Golgi complex factor GRIP and coiled-coil domain containing 2 (GCC2), which leads to decreased replication and virus titers of herpes simplex virus infection [99]. Another study showed that losing the essential component of the adhesive complex stromal antigen 2 (STAG2) in HT-29 cells affects the DNA sensing pathway of cGAS-STING and significantly reduces the replication of rotavirus in the cells [100]. These findings could become potential targets for disease-resistance breeding through genome editing technology.

## Figures and Tables

**Table 1:** ASFV proteins and immunomodulation tactics.

Viral Protein	Functions	Pathway	References
pC129R	Target Cyclic GMP- AMP To Inhibit the cGAS-STING Signaling Pathway	cGAS-STING	[13]
pEP364R	Target Cyclic GMP- AMP To Inhibit the cGAS-STING Signaling Pathway	cGAS-STING	[13]
pA137R	Inhibited the nuclear import of IRF3	cGAS-STING/Ubiquitination	[14]
pM1249L	Suppress phosphorylation of TBK1 and degrading IRF3	cGAS-STING	[15]
p17	Inhibits cGAS-STING signaling pathway through interacting with STING	cGAS-STING	[16]
pMGF 505-7R	Inhibit the translocation of IRF3 to the nucleus	cGAS-STING/Ubiquitination	[11]
pMGF 360-11L	Inhibits IL-1β, IL-6, and IFN-β secretion	cGAS-STING	[17]
p14.5	Blocks IRF3 phosphorylation	cGAS-STING	[18]
MGF 505-11R	Binds to STING and promotes its degradation through the lysosomal and autophagy mechanisms	cGAS-STING	[19]
pD345L	Suppress NF-κB signaling by hindering the activity of the IKK kinase	NF-κB	[20]
pI226R	Suppresses the stimulation of NF-κB	NF-κB	[21]
pF317L	Bound with IκB kinase β (IKKβ) and hindered its phosphorylation,	NF-κB	[22]
pA528R	InterruptNF-κB, inhibit downstream promoters, phosphorylation of NF-κB p65,	NF-κB	[23]
pI215L	encode the ubiquitin-conjugating enzyme making	Ubiquitination	[24]
pE199L	Promotes cell autophagy through the interaction of PYCR2	Ubiquitination	[25]
pK205R	Activates autophagy and the NF-κB signaling pathway	Ubiquitination	[26]
pA179L	Anti-apoptotic agent	Apoptosis	[27,28,29]
pA224L	Blocks the activation of caspase-3 and enhances the ability of cells to survive	Apoptosis	[30]
pEP153R	Prevent apoptosis via activating the p53 and caspase 3 pathways	Apoptosis	[31,32]
pDP71L	Dephosphorylation of eIF2α and deactivation of the proapoptotic CHOP factor	Apoptosis	[33]
pA238L	Inhibitor of NF-κB pathway	NF-κB	[34]
p54	Enhances apoptosis induction	Apoptosis	[35]
pE248R	Inhibit the expression of STING protein	cGAS-STING	[36]
pS273R	Interfere with the interaction between IKK and STING.	NF-κB	[37]

## Data Availability

Not applicable.
